# Root biomass and exudates link plant diversity with soil bacterial and fungal biomass

**DOI:** 10.1038/srep44641

**Published:** 2017-04-04

**Authors:** Nico Eisenhauer, Arnaud Lanoue, Tanja Strecker, Stefan Scheu, Katja Steinauer, Madhav P. Thakur, Liesje Mommer

**Affiliations:** 1 German Centre for Integrative Biodiversity Research (iDiv) Halle-Jena-Leipzig, Deutscher Platz 5e, 04103 Leipzig, Germany; 2 Institute of Biology, Leipzig University, Johannisallee 21, 04103 Leipzig, Germany; 3 Université François Rabelais de Tours. EA 2106 Plant Biotechnology and Biomolecules, 31 Avenue Monge, F-37200 Tours, France; 4 Georg August University Göttingen, J.F. Blumenbach Institute of Zoology and Anthropology, Berliner Str. 28, 37073 Göttingen, Germany; 5 Plant Ecology and Nature Conservation group, Wageningen University, PO Box 47, 6700 AA Wageningen, The Netherlands

## Abstract

Plant diversity has been shown to determine the composition and functioning of soil biota. Although root-derived organic inputs are discussed as the main drivers of soil communities, experimental evidence is scarce. While there is some evidence that higher root biomass at high plant diversity increases substrate availability for soil biota, several studies have speculated that the quantity and diversity of root inputs into the soil, i.e. though root exudates, drive plant diversity effects on soil biota. Here we used a microcosm experiment to study the role of plant species richness on the biomass of soil bacteria and fungi as well as fungal-to-bacterial ratio *via* root biomass and root exudates. Plant diversity significantly increased shoot biomass, root biomass, the amount of root exudates, bacterial biomass, and fungal biomass. Fungal biomass increased most with increasing plant diversity resulting in a significant shift in the fungal-to-bacterial biomass ratio at high plant diversity. Fungal biomass increased significantly with plant diversity-induced increases in root biomass and the amount of root exudates. These results suggest that plant diversity enhances soil microbial biomass, particularly soil fungi, by increasing root-derived organic inputs.

Plant diversity is a significant driver of multiple ecosystem functions, such as plant biomass production^1^, and carbon sequestration^2^. Recent studies in controlled experiments indicate that soil microorganisms are closely tied to plant diversity effects on multiple ecosystem functions^3^,^4^,^5^,^6^. Accordingly, the functional composition of soil microbial communities changes with plant diversity, e.g., by increasing the relative biomass of fungi over bacteria^3^,^7^ and altering soil enzyme activities^3^,^7^. Notably, evidence from natural ecosystems for significant relationships between plant diversity and microbial community composition and functions is mixed^8^,^9^, partly because both plant diversity and soil microorganisms are influenced by a plethora of abiotic and biotic determinants^10^,^11^. This is why we refer to the results of controlled plant diversity experiments in the following.

Standing root biomass is known to increase with plant diversity^12^,^13^,^14^, and enhanced root biomass is likely to increase the resource availability for soil microbial communities^15^ *via* altered root turnover and/or root exudation. While root turnover has been shown to decline with plant diversity^16^,^17^, knowledge of effects of plant diversity on root exudation is scarce (but see e.g., ref. 18). In fact, several studies have speculated that the quantity and diversity of root inputs into the soil, i.e. though root exudates, drive plant diversity effects on soil biota. The quantity and quality of root exudates released into the soil is context-dependent, e.g., differ with plant species, functional dissimilarity among plant species^10^, plant age^19^, and soil conditions^20^. Root exudates have been shown to play a significant role in numerous ecosystem functions, such as nutrient availability^21^ and cycling^22^. The biological activity of root exudates might vary with both the structure of the compounds and their local concentration in the rhizosphere^23^, while synergistic effects with other root exudates might alter this activity, subsequent effects on soil microbial communities, as well as interactions among plants and rhizosphere microbial communities^21^. Thus, root exudates are likely to shift with changing biotic and abiotic conditions along the plant diversity gradient^3^,^24^.

Evidence for root exudate-mediated effects of plant diversity on soil microbial communities stems from indirect approaches, such as ascribing unexplained variance in linear regression models^25^ or structural equation models^26^ to root exudates. Recently, using a ^13^C labelling approach, Lange *et al*.^6^ provided experimental evidence for the significant role of root exudates in fuelling soil microorganisms. They showed that plant diversity increases the transfer of ^13^C from plants to soil microbial fatty acids. Moreover, Sauheitl *et al*.^27^ found that the composition, but not the amount, of amino acids changed along a plant diversity gradient. However, it remains unclear if soil microbial communities depend on plant diversity-driven variations in root biomass and composition^28^, root diversity^29^, quantity and diversity of root exudates^6^,^26^, or their combination.

Here we used a microcosm experiment to investigate effects of plant diversity on the biomass of soil bacteria and fungi. We hypothesized that plant diversity effects are mediated by changes in root biomass^25^,^28^,^30^ and the diversity and quantity of root exudates^6^,^25^,^29^. Species-specific root biomass was determined *via* quantitative PCR, and the diversity and quantity of root exudates were assessed *via* high pressure liquid chromatography (HPLC).

## Results

### Plant biomass

**Figure 1:**
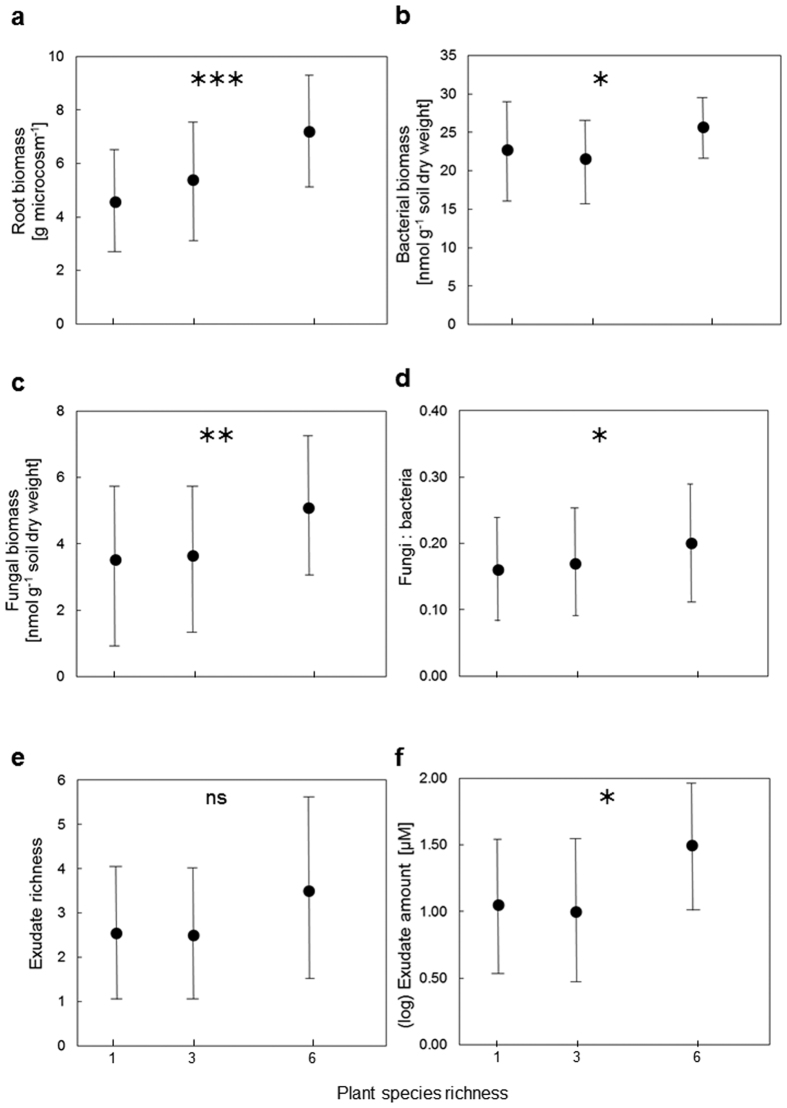
Plant diversity effects on root biomass, soil microbial properties, and root exudates. Root biomass [g microcosm^−1^] (**a**), bacterial biomass [nmol g^−1^ soil dry weight] (**b**), fungal biomass [nmol g^−1^ soil dry weight] (**c**), ratio between fungal and bacterial biomass (**d**), root exudate diversity [richness of different compounds] (**e**), and root exudate amount [μM] as the sum of the most abundant compounds fumaric acid, 4-hydroxybenzoic acid, p-coumaric acid, and vanillic acid; see main text for details (**f**) as affected by plant species richness. Given are means with 95% confidence intervals. ***p ≤ 0.001; **p ≤ 0.01; *p ≤ 0.05 (see Table 1 for details).

**Figure 2:**
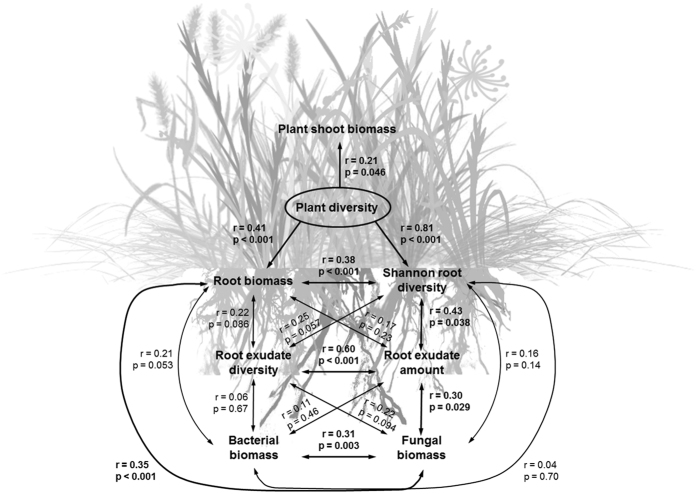
Relationships between plant diversity, root biomass and exudates, and soil microorganisms. Correlations among plant diversity (experimental treatment; 1, 3, or 6 plant species) and plant shoot biomass, root biomass (both [g microcosm^−1^]), Shannon diversity of roots, root exudate diversity [richness of different compounds], amount of root exudates (log total of the most frequent compounds fumaric acid, 4-hydroxybenzoic acid, p-coumaric acid, and vanillic acid [μM]; see main text for details), soil bacterial biomass, and soil fungal biomass (both [nmol g^−1^ soil dry weight]). One-headed arrows indicate treatment effects on response variables, while double-headed arrows indicate correlations. Significant relationships (*p* < 0.05) are given in bold; *r* stands for correlation coefficient. Note that results of structural equation modelling are not shown due to low replication number, but confirmed the significant relationship between the amount of root exudates and fungal biomass (*p* = 0.046).

Shoot and root biomass increased significantly with increasing plant species richness (Table 1; Figs 1 and 2). However, belowground net biodiversity effect (NE), complementarity effect (CE), and selection effect (SE) did not differ significantly between plant mixtures with 3 and 6 species (Table 1).

### Root exudates

In root exudates, we identified a total of 15 plant-derived compounds, i.e., fumaric acid, chlorogenic acid, 4-hydroxybenzoic acid, vanillic acid, syringic acid, 4-hydroxybenzaldehyde, vanillin, polydatin, p-coumaric acid, *t*-2-methoxycinnamic acid, phenylacetic acid, coumarin, a flavone-type compound, 2 quercetin glycoside derivatives, and a benzoate ester. Chlorogenic acid is a root exudate marker of *P. pratense*, polydatin of *R. acetosa*, and coumarin of *A. odoratum*. Most frequent compounds were used for quantification of root exudates as they were detected in many samples, i.e., fumaric acid (48 of 54 samples), vanillic acid (38/54), p-coumaric acid (14/54), and 4-hydroxybenzoic acid (9/54). The amounts of these four compounds were summed up and used as index of total root exudate amount. This index of the total amount of root exudates represented the amount of all 15 compounds very well (*r* = 0.63, *p* < 0.001).

Root exudate diversity and the amount of root exudates were significantly positively correlated (Fig. 2). The amount of root exudates increased significantly with increasing plant diversity, while exudate diversity did not (Table 1; Figs 1 and 2). While root exudate diversity only tended to increase with root biomass and Shannon diversity of roots (which strongly depended on the experimental treatment plant species richness of necessity), the amount of root exudates was significantly positively correlated with the Shannon diversity of roots, but not with root biomass (Fig. 2).

### Bacterial and fungal biomass

The biomass of bacteria and fungi as well as the ratio between fungi and bacteria increased significantly with increasing plant species richness (Table 1; Fig. 1). The biomass of bacteria and fungi were significantly positively correlated (Fig. 2). Bacterial biomass tended to increase with increasing root biomass, while the biomass of fungi increased significantly with increasing root biomass. Further, fungal biomass (Fig. 2) and the ratio between fungi and bacteria (*r* = 0.32, *p* = 0.02) increased significantly with increasing amount of root exudates. Fungal biomass (Fig. 2) and the ratio between fungi and bacteria (*r* = 0.25, *p* = 0.057) also tended to increase with exudate diversity.

## Discussion

The present microcosm experiment mirrors field plant diversity experiments by showing increased above-1,31 and belowground^13^,^14^ plant biomass with increasing plant diversity. Consistent with field experiments^26^,^32^, soil microbial biomass increased with plant diversity, and soil microbial communities shifted along the plant diversity gradient towards more fungal-dominated communities^6^,^33^. While our results confirm previous experiments that reported plant biomass-mediated effects of plant diversity on soil microbial biomass^32^, the present study provides one of the first empirical evidences that this plant diversity effect could be driven –at least in part– by changes in plant root exudation.

Plants often select their rhizobiomes *via* root exudation^21^. Indeed, an elevated amount, and to a lower extent diversity, of root exudates at high plant diversity shifted fungal-to-bacterial ratio in the present study. A recent laboratory experiment also suggests that variations in the diversity and stoichiometry of root exudates can alter soil microbial communities and functions as well as influence the relationship between plant diversity and microbial communities^29^. However, it is important to note that soil microbial communities can also influence the amount and composition of root exudates^34^. The marginally significant positive relationship between the diversity of root exudates and root biomass found in the present study may have been due to larger surface area of the roots^35^,^36^ and a higher Shannon diversity of roots (with the latter being directly related to the experimental plant diversity gradient).

The investigation of root exudates is challenging, and we had to accept some limitations of our approach. First of all, we were able to identify only a fraction of the compounds detected in the HPLC; nevertheless we used identified plant products only, because organic compounds in the soil will always contain soil microbial products^30^ that were not in the focus of this study. Thus, the measures of root exudate amount and diversity should be regarded as proxies representing relative differences among experimental treatments rather than absolute measures. Despite those caveats, the present study provides empirical evidence for the significant role of root exudates in linking above- and belowground communities and the diversity of plant communities with the functional composition of soil microbial communities^12^,^22^ stimulating future work on the mechanisms of rhizosphere interactions^26^,^28^,^33^.

Ecosystem services, such as soil carbon storage^6^ and soil aggregate stability^37^, are known to be driven by soil microorganisms and contingent upon plant diversity. The fungal-to-bacterial biomass ratio is a powerful predictor of soil ecosystem functioning, varies with biotic and abiotic environmental factors^3^,^38^, and thus is likely to be affected by root litter and organic compounds exudated by roots into the rhizosphere^6^. Hooper *et al*.^29^ proposed that resource heterogeneity determines the composition and diversity of soil microorganisms, although experimental evidence is scarce^39^. The present study is one of the first indicating that the diversity of roots and root exudates, i.e., the heterogeneity of resources of soil microorganisms, indeed influence the composition and biomass of soil microbial communities, although the quantity of roots and root exudates were the most significant determinants of fungal biomass and the functional composition of soil microbial communities (fungal-to-bacterial ratio). Integrative approaches combining chemical and microbial ecology^40^,^41^ are needed to improve mechanistic understanding of aboveground-belowground linkages. Understanding the links between root diversity, root biomass production, root exudates, and soil microorganisms and how they are affected by plant diversity is important for maintaining ecosystem service provision at times of globally declining diversity.

## Material and Methods

### Experimental set-up

Three different plant diversity levels were established using a pool of six species: monocultures (N = 54), 3- (N = 24), and 6-species mixtures (N = 15). Plant species were selected based on their functional dissimilarity in spatial resource use according to Ebeling *et al*.^42^: the grasses *Festuca rubra, Phleum pratense, Anthoxanthum odoratum* and the forbs *Plantago lanceolata, Centaurea jacea, Rumex acetosa*. This trait-based approach was chosen as our main hypothesis, of plant diversity effects on soil microorganisms being mediated by changes in root biomass and the diversity and quantity of root exudates, required functional dissimilarity among plant species. The initial design of the experiment included an orthogonal manipulation of plant diversity and decomposer diversity (see Supporting Information). However, as the decomposer treatments could not be successfully implemented, and decomposers could not be retrieved at the end of the experiment (probably due to a dry period during the experiment; microcosms were watered manually, see below), we focus on plant species richness effects in the present study and regard the decomposer treatments as potential source for variability in the dataset (e.g., due to potential nutrient effects of decomposing soil animals).

Microcosms consisted of PVC tubes (inner diameter 10 cm, height 20 cm) sealed with PVC lids at the bottom. Each microcosm was filled with 1.6 kg fresh, sieved (4 mm), and defaunated (heating and drying the soil^43^) soil (pH 8.1, carbon concentration 4.6%, nitrogen concentration 0.3%, C-to-N ratio 15.7; water content 14%) from the grassland field site of the Jena Experiment^44^. The soil was washed to reduce potential effects of nutrients released by the defaunation process. Seedlings (at least 3 cm in height) of six pre-grown plants (also grown in the defaunated and washed soil mentioned above) were transplanted into microcosms in a defined pattern to enhance interspecific interactions; i.e., plant individuals always had neighbours from other plant species in mixtures. During the experiment, microcosms were irrigated with deionized water with the amount increasing from 50 ml/2d to 50–100 ml/day later in the experiment. Microcosms received the same amount of water because variations in soil water content may co-determine plant diversity effects on soil microorganisms^24^. No nutrients were added to during the experiment. Microcosms were incubated in a climate chamber (day/night = 16/8 h and 22/18 °C ± 3.5 °C; PAR 400 μE) for 104 days.

### Harvest and laboratory analyses

Root exudates were sampled directly before the harvest with micro-suction cups (Rhizon Mom 10 cm, Rhizosphere Research Products, Wageningen, Netherlands) and stored until analyses at −20 °C (see Supporting Information for more details). Root exudate solutions (10 ml) were filtered through a 0.22 μm filter (Millipore, Bedford, MA, USA), acidified to pH 2 with 1 N HCl, and partitioned against 5 ml ethyl acetate on an orbital shaker at 290 rpm (Bühler, Hechingen, Germany) for 1 h at room temperature. The organic phase was reduced to dryness under nitrogen and the residue taken up in 100 μl of methanol for high pressure liquid chromatography (HPLC) analyses (see ref. 40; Supporting Information). We only considered compounds that could be ascribed doubtlessly to plant products among many peaks in the HPLC chromatograms, because soluble organic compounds in the soil can contain root exudates as well as soil microbial products^30^. Thus, our index of root exudate diversity can only be a proxy measure of true diversity of root exudates. Due to the laborious analysis, root exudates could be quantified in 54 microcosms (29, 16, and 9 replicates in monocultures, 3- and 6-species treatments, respectively).

As given above, we used the most frequent compounds for quantification of root exudates: fumaric acid, vanillic acid, p-coumaric acid, and 4-hydroxybenzoic acid. We used this approach as we had to focus on well quantifiable compounds. Every analytical method involves a Limit Of Quantification (LOQ) and a Limit of Detection (LOD). “Not quantified” means that the detected signal does not allow a robust quantification, but not that the compound is absent. Here, the amount of detected root exudates was very low, which still represents a limit for the study of belowground signalling molecules^40^, probably due to the intrinsic limitations of the sampling method (micro-suction cups) and the high turnover of root exudates. Thus, the sum of those four root exudates provides a stoichiometric-based calculation of the total amount of root exudates. While we limited the quantification of root exudates to the four compounds that were above the LOQ, other compounds were only detectable (above LOD) but not quantifiable (below LOQ) and thus were only used for the assessment of root exudate diversity.

We restricted our study to the water-soluble fraction of root exudates as defined in Bais *et al*.^45^ and Baetz & Martinoa^46^. These exudates cover plant secondary metabolites affecting soil microorganisms. Although unidentified peaks observed during HPLC analyses indicate that other compounds, such as phenolic acids, were present in the root exudate solution and could also contribute to the mediation of plant diversity effects on soil microorganisms, the characterization of the root exudates remains a challenge. Novel analytical platforms likely will offer unprecedented opportunities to unravel the complex composition of root exudates in future studies^40^.

The root exudates analyzed in the present study were indubitably identified by the use of pure external standards purchased from Sigma-Aldrich (St Louis, MI, USA). 4-hydroxybenzoic acid, p-coumaric acid, and vanillic acids belong to the class of phenolic compounds. This class of plant secondary metabolites derives from the shikimate pathway and phenylpropanoid metabolism and has been extensively studied as plant allelochemicals. They are commonly found in natural soils^47^,^48^, and there are known as plant root exudates playing a role in root inhibition^49^, plant defense^50^,^51^, and in the attraction of some soil-borne microorganisms^52^. Fumaric acid is an organic acid that is a key intermediate in the tricarboxylic acid cycle and plays a role in many other plant metabolic processes. Organic acids are commonly detected in the rhizosphere. Their presence in root exudation have been associated with nutrient deficiencies, exposure to toxic cations and anoxia^53^, and contribute to the stimulation of microbial activity^54^. Previous studies using sterile hydroponic plants have shown that the described molecules (belonging to organic and phenolic acids) are part of plant root exudation; however, often to a lower extent compared to real soil-root systems. The study of root exudates as signalling molecules of belowground plant interactions necessarily implies to work with microorganisms at the root-soil interface, which may degrade these compounds sooner or later.

Plants shoot material was cut at the soil surface, dried (70 °C, 72 h), and weighed. For soil microbial analyses, three soil cores (1 cm diameter, 10 cm deep) were taken per microcosm, pooled, and frozen at −20 °C until analysis. For molecular species-specific root quantification, one subsample was taken in the centre of the microcosm to include roots of all plant species (0–10 cm: 1 cm diameter, 10–20 cm: 2.5 cm diameter). Roots were washed (1 mm-sieve), thoroughly mixed, and 100 mg of fresh root material was stored at −80 °C until DNA extraction. The remaining soil in the microcosm was washed, and roots were dried and weighed like described above.

For preparation of reference standards for quantitative analysis of species-specific root biomass with real-time PCR, hand-made mixed ratios of all 6 species (1:1:1:1:1:1) were prepared from roots originating from the respective monocultures. The DNeasy Plant Mini Kit (Qiagen, Venlo, Netherlands) was used for DNA isolation. For qPCR, species-specific primers of *A. odoratum* and *F. rubra* were used as published in refs 12, 55; primers for *P. lanceolata, R. acetosa, P. pratense*, and *C. jacea* were developed similar to the protocol described in ref. 55. The qPCR plates (96 wells system) were run per species in triplicates, including 4 reference standards. We used the following protocol for the qPCR with HOT FIREPol EvaGreen (Solis BioDyne) with an addition of 0.94 μM MgCl_2_, 1 ng of genomic DNA for all species except for *Plantago* for which 7.5 ng of genomic DNA was used, a primer concentration of 120 nM for *Festuca, Plantago, Rumex,* and *Phleum* and 60 nM for *Anthoxanthum* and *Centaurea* and in a total volume of 20 μl. qPCR reactions were performed on a CFX96 Real-Time system (Biorad). The qPCR reaction conditions were as follows: 15 min at 92 °C, followed by 45 cycles of 20 s at 95 °C, 30 s at 62 °C, and 15 s at 72 °C and finally 42 cycles of 5 s at 75 °C with an increase of 0.5 °C per cycle (melting curve). For the calculation of relative species abundances see Mommer *et al*.^55^. Root DNA analyses could only be performed for 27 plant species mixtures (17 3-species mixtures and 10 6-species treatments) due to logistic constraints as a consequence of the plethora of different laborious analyses.

To determine soil microbial community composition and biomass at the end of the experiment, phospholipid fatty acid (PLFA) analysis was used. PLFA extraction was done following the method of ref. 56, based on the method of ref. 57, and PLFAs were assigned to Gram-positive bacteria, Gram-negative bacteria, fungi, and unidentified microorganisms.

### Calculations and statistical analyses

Species-specific root biomass allowed performing additive partitioning^58^ for calculating belowground net biodiversity effect (NE), complementarity effect (CE), and selection effect (SE). Further, the Shannon diversity index of root biomass was calculated to include the relative abundances of different plant species in the belowground plant diversity measure. General Linear Models were used to test plant species richness effects on shoot biomass, root biomass, Shannon diversity of roots, complementarity effect, selection effect, net biodiversity effect, root exudate richness, root exudate amount, bacterial biomass, fungal biomass, and the ratio between fungal and bacterial biomass (Table 1). Further, linear regressions were used to test the relationships between plant species richness and plant shoot biomass, root biomass, and soil microbial variables as well as between different response variables (Supporting Information). Statistical analyses were performed using Statistica 10 (Statsoft). Given the rather low number of replicates for several variables (root exudates and species-specific root biomass), structural equation modeling could not be used to test our hypotheses^59^. We ran SEM and piecewise SEM^60^, but the best model did not meet the criteria based on Fisher’s C statistic (Fisher’s C = 66.93, p-value = 0.009, AIC = 168.93, AICc = −27.51), which was most likely due to a low number of replicates (n = 25; i.e., in 25 microcosms both species-specific root biomass and root exudates were analyzed). Despite the flexibility of using piecewise SEM for lower number of replicates, our model was still invalid due to several variables and paths. Thus, despite the targeted experimental design, most of the present results are based on correlations, which may not necessarily imply causalities. Notably, however, results of structural equation modeling confirmed the significant relationship between the amount of root exudates and fungal biomass (*p* < 0.046).

### Data accessibility

Data available from the Dryad Digital Repository: http://dx.doi.org/10.5061/dryad.nj3c0.

## Additional Information

**How to cite this article**: Eisenhauer, N. *et al*. Root biomass and exudates link plant diversity with soil bacterial and fungal biomass. *Sci. Rep.* **7**, 44641; doi: 10.1038/srep44641 (2017).

**Publisher's note:** Springer Nature remains neutral with regard to jurisdictional claims in published maps and institutional affiliations.

## Supplementary Material

Supplementary Information

## Figures and Tables

**Table 1:** GLM table of effects of plant diversity (1, 3, and 6 plant species per microcosm) on plant shoot and root biomass [g microcosm^−1^], Shannon diversity of root biomass, root complementarity effect [g microcosm^−1^], root selection effect [g microcosm^−1^], root net biodiversity effect [g microcosm^−1^], exudate richness [# of compounds], exudate amount [μM], soil bacterial biomass [nmol g^−1^ soil dry weight], fungal biomass [nmol g^−1^ soil dry weight], and ratio between fungal and bacterial biomass.

Variable	1 species	3 species	6 species	df	fit	*F*	*p*
**Shoot biomass**	**2.48 ± 0.86**	**2.53 ± 0.81**	**3.05 ± 0.89**	**1**, **90**	**l**	**4.10**	**0.046**
**Root biomass**	**4.57 ± 1.71**	**5.40 ± 2.34**	**7.19 ± 2.30**	**1**, **90**	**l**	**17.98**	**<0.001**
**Shannon diversity roots**	**0.00 ± 0.00**	**0.86 ± 0.18**	**1.35 ± 0.14**	**1**, **88**	**l**	**699.5**	**<0.001**
Root complementarity effect	nc	−0.65** ± **1.15	−0.39** ± **0.66	1, 32	c	1.80	0.187
Root selection effect	nc	1.82** ± **3.50	3.30** ± **2.62	1, 32	c	0.56	0.458
***Root net biodiversity effect***	***nc***	***1.18***** ± *****2.96***	***2.92***** ± *****2.76***	***1**, **32***	***c***	***3.11***	***0.086***
Exudate richness	2.55** ± **1.46	2.50** ± **1.42	3.50** ± **2.17	2, 51	c	1.58	0.215
**Exudate amount (log10)**	**1.05 ± 0.51**	**1.00 ± 0.54**	**1.50 ± 0.41**	**2**, **51**	**c**	**3.29**	**0.045**
**Bacterial biomass**	**22.70 ± 6.54**	**21.58 ± 5.84**	**25.70 ± 3.84**	**2**, **85**	**c**	**3.78**	**0.027**
**Fungal biomass**	**3.52 ± 2.44**	**3.64 ± 2.24**	**5.08 ± 2.24**	**1**, **86**	**l**	**9.39**	**0.003**
**Fungi: bacteria**	**0.16 ± 0.08**	**0.17 ± 0.08**	**0.20 ± 0.09**	**1**, **84**	**l**	**4.46**	**0.038**

Given are means ± standard deviation, degrees of freedom (df), fit of the factor plant species richness, and *F*- and *p*-values.

Nc: not possible to calculate because monocultures are used as reference for additive partitioning calculations; c: categorical factor; l: linear variable; significant plant species richness effects (*p* < 0.05) are given in bold.

## References

[b1] Reich P. B. . Impacts of biodiversity loss escalate through time as redundancy fades. Science 336, 589–92 (2012). 22556253 10.1126/science.1217909

[b2] Cong W.-F. . Plant species richness promotes soil carbon and nitrogen stocks in grasslands without legumes. J. Ecol. 102, 1163–1170 (2014).

[b3] Chung H., Zak D. R., Reich P. B. & Ellsworth D. S. Plant species richness, elevated CO 2, and atmospheric nitrogen deposition alter soil microbial community composition and function. Glob. Chang. Biol. 13, 980–989 (2007).

[b4] Schnitzer S. . Soil microbes drive the classic plant diversity-productivity pattern. Ecology 92, 296–303 (2011). 21618909 10.1890/10-0773.1

[b5] Kulmatiski A., Beard K. H. & Heavilin J. Plant-soil feedbacks provide an additional explanation for diversity-productivity relationships. Proc. Biol. Sci. 279, 3020–3026 (2012). 22496190 10.1098/rspb.2012.0285 PMC3385476

[b6] Lange M. . Plant diversity increases soil microbial activity and soil carbon storage. Nat. Commun. 6, 6707 (2015). 25848862 10.1038/ncomms7707

[b7] Steinauer K. . Plant diversity effects on soil microbial functions and enzymes are stronger than warming in a grassland experiment. Ecology 96, 99–112 (2015). 26236895 10.1890/14-0088.1

[b8] Eisenhauer N. . Positive relationship between herbaceous layer diversity and the performance of soil biota in a temperate forest. Soil Biol. Biochem. 43, 462–465 (2011).

[b9] Prober S. M. . Plant diversity predicts beta but not alpha diversity of soil microbes across grasslands worldwide. Ecol. Lett. 18, 85–95 (2015). 25430889 10.1111/ele.12381

[b10] Eisenhauer N. . Biodiversity-ecosystem function experiments reveal the mechanisms underlying the consequences of biodiversity change in real world ecosystems. J. Veg. Sci. 27, 1061–1070 (2016).

[b11] Grace J. B. . Integrative modelling reveals mechanisms linking productivity and plant species richness. Nature 529, 1–10 (2016). 10.1038/nature16524 26760203

[b12] Mommer L. . Unveiling below-ground species abundance in a biodiversity experiment: a test of vertical niche differentiation among grassland species. J. Ecol. 98, 1117–1127 (2010).

[b13] Mueller K., Tilman D., Fornara D. & Hobbie S. Root depth distribution and the diversity-productivity relationship in a long-term grassland experiment. Ecology 94, 787–793 (2013).

[b14] Ravenek J. M. . Long-term study of root biomass in a biodiversity experiment reveals shifts in diversity effects over time. Oikos 123, 1528–1536 (2014).

[b15] Philippot L., Raaijmakers J. M., Lemanceau P. & van der Putten W. H. Going back to the roots: the microbial ecology of the rhizosphere. Nat. Rev. Microbiol. 11, 789–799 (2013). 24056930 10.1038/nrmicro3109

[b16] Fornara D. A., Tilman D. & Hobbie S. E. Linkages between plant functional composition, fine root processes and potential soil N mineralization rates. J. Ecol. 97, 48–56 (2009).

[b17] Mommer L. . Diversity effects on root length production and loss in an experimental grassland community. Funct. Ecol. 29, 1560–1568 (2015).

[b18] De Deyn G. B., Quirk H., Oakley S., Ostle N. J. & Bardgett R. D. Increased plant carbon translocation linked to overyielding in grassland species mixtures. PLoS One 7, e45926 (2012). 23049893 10.1371/journal.pone.0045926 PMC3457971

[b19] Jones D. L., Nguyen C. & Finlay R. D. Carbon flow in the rhizosphere: carbon trading at the soil–root interface. Plant Soil 321, 5–33 (2009).

[b20] Boone R. D., Nadelhoffer K. J., Canary J. D. & Kaye J. P. Roots exert a strong influence on the temperature sensitivity of soil respiration. Nature 396, 570–572 (1998).

[b21] Mommer L., Kirkegaard J. & van Ruijven J. Root–Root Interactions: Towards A Rhizosphere Framework. Trends Plant Sci. 21, 209–217 (2016). 26832947 10.1016/j.tplants.2016.01.009

[b22] Keiluweit M. . Mineral protection of soil carbon counteracted by root exudates. Nat. Clim. Chang. 5, 588–595 (2015).

[b23] Kuzyakov Y. & Blagodatskaya E. Microbial hotspots and hot moments in soil: Concept & review. Soil Biol. Biochem. 83, 184–199 (2015).

[b24] Lange M. . Biotic and abiotic properties mediating plant diversity effects on soil microbial communities in an experimental grassland. PLoS One 9, e96182 (2014). 24816860 10.1371/journal.pone.0096182 PMC4015938

[b25] Eisenhauer N. . Plant diversity effects on soil microorganisms support the singular hypothesis. Ecology 91, 485–96 (2010). 20392013 10.1890/08-2338.1

[b26] Eisenhauer N. . Plant diversity effects on soil food webs are stronger than those of elevated CO2 and N deposition in a long-term grassland experiment. Proc. Natl. Acad. Sci. 110, 6889–6994 (2013). 23576722 10.1073/pnas.1217382110 PMC3637779

[b27] Sauheitl L., Glaser B., Dippold M., Leiber K. & Weigelt A. Amino acid fingerprint of a grassland soil reflects changes in plant species richness. Plant Soil 334, 353–363 (2010).

[b28] Spehn E., Joshi J., Schmid B., Alphei J. & Körner C. Plant diversity effects on soil heterotrophic activity in experimental grassland ecosystems. Plant Soil 224, 217–230 (2000).

[b29] Hooper D. U. . Interactions between Aboveground and Belowground Biodiversity in Terrestrial Ecosystems: Patterns, Mechanisms, and Feedbacks. Bioscience 50, 1049 (2000).

[b30] Milcu A., Partsch S., Scherber C., Weisser W. & Scheu S. Earthworms and legumes control litter decomposition in a plant diversity gradient. Ecology 89, 1872–1882 (2008). 18705374 10.1890/07-1377.1

[b31] Cardinale B. J. . Impacts of plant diversity on biomass production increase through time because of species complementarity. Proc. Natl. Acad. Sci. USA 104, 18123–18128 (2007). 17991772 10.1073/pnas.0709069104 PMC2084307

[b32] Thakur M. P. . Plant diversity drives soil microbial biomass carbon in grasslands irrespective of global environmental change factors. Glob. Chang. Biol. 21, 4076–4085 (2015). 26118993 10.1111/gcb.13011

[b33] Eisenhauer N., Migunova V. D., Ackermann M., Ruess L. & Scheu S. Changes in plant species richness induce functional shifts in soil nematode communities in experimental grassland. PLoS One 6, e24087 (2011). 21909412 10.1371/journal.pone.0024087 PMC3164708

[b34] Meier I. C., Avis P. G. & Phillips R. P. Fungal communities influence root exudation rates in pine seedlings. FEMS Microbiol. Ecol. 83, 585–95 (2013). 23013386 10.1111/1574-6941.12016

[b35] Subedi K. D., Ma B. L. & Liang B. C. New method to estimate root biomass in soil through root-derived carbon. Soil Biol. Biochem. 38, 2212–2218 (2006).

[b36] Wurst S., Wagenaar R., Biere A. & Van der Putten W. H. Microorganisms and nematodes increase levels of secondary metabolites in roots and root exudates of Plantago lanceolata. Plant Soil 329, 117–126 (2010).

[b37] Rillig M. C. & Mummey D. L. Mycorrhizas and soil structure. New Phytol. 171, 41–53 (2006). 16771981 10.1111/j.1469-8137.2006.01750.x

[b38] de Vries F. T. . Soil food web properties explain ecosystem services across European land use systems. Proc. Natl. Acad. Sci. U. S. A. 110, 14296–301 (2013). 23940339 10.1073/pnas.1305198110 PMC3761618

[b39] Bardgett R. & Wardle D. Aboveground-belowground linkages: biotic interactions, ecosystem processes, and global change. (Oxford University Press, New York, USA, 2010).

[b40] van Dam N. M. & Bouwmeester H. J. Metabolomics in the Rhizosphere: Tapping into Belowground Chemical Communication. Trends Plant Sci. 21, 256–265 (2016). 26832948 10.1016/j.tplants.2016.01.008

[b41] Powell J. R., Craven D. & Eisenhauer N. Recent trends and future strategies in soil ecological research-Integrative approaches at Pedobiologia. Pedobiologia 57, 1–3 (2014).

[b42] Ebeling A. . A trait-based experimental approach to understand the mechanisms underlying biodiversity–ecosystem functioning relationships. Basic Appl. Ecol. 15, 229–240 (2014).

[b43] Huhta V., Wright D. H. & Coleman D. C. Characteristics of defaunated soil. I: A comparison of three techniques applied to two different forest soils. Pedobiologa 33, 415–424 (1989).

[b44] Roscher C., Schumacher J. & Baade J. The role of biodiversity for element cycling and trophic interactions: an experimental approach in a grassland community. Basic Appl. Ecol. 121, 107–121 (2004).

[b45] Bais H. P., Weir T. L., Perry L. G., Gilroy S. & Vivanco J. M. The role of root exudates in rhizosphere interactions with plants and other organisms. Annu. Rev. Plant Biol. 57, 233–66 (2006). 16669762 10.1146/annurev.arplant.57.032905.105159

[b46] Baetz U. & Martinoia E. Root exudates: the hidden part of plant defense. Trends Plant Sci. 19, 90–8 (2014). 24332225 10.1016/j.tplants.2013.11.006

[b47] Whitehead D. Identification of p-hydroxybenzoic, vanillic, p-coumaric and ferulic acids in soils. Nature 202, 417–418 (1964). 14152852 10.1038/202417a0

[b48] Wang T., Yang T. & Chuang T. Soil phenolic acids as plant growth inhibitors. Soil Sci. 103, 239–246 (1967).

[b49] Weir T. L., Park S. W. & Vivanco J. M. Biochemical and physiological mechanisms mediated by allelochemicals. Curr. Opin. Plant Biol. 7, 472–479 (2004). 15231272 10.1016/j.pbi.2004.05.007

[b50] Lanoue A. . De novo biosynthesis of defense root exudates in response to Fusarium attack in barley. New Phytol. 185, 577–588 (2010). 19878462 10.1111/j.1469-8137.2009.03066.x

[b51] Cannesan M. A. . Association between border cell responses and localized root infection by pathogenic Aphanomyces euteiches. Ann. Bot. 108, 459–469 (2011). 21807690 10.1093/aob/mcr177 PMC3158693

[b52] Badri D. V., Chaparro J. M., Zhang R., Shen Q. & Vivanco J. M. Application of natural blends of phytochemicals derived from the root exudates of arabidopsis to the soil reveal that phenolic-related compounds predominantly modulate the soil microbiome. J. Biol. Chem. 288, 4502–4512 (2013). 23293028 10.1074/jbc.M112.433300 PMC3576057

[b53] Ryan P., Delhaize E. & Jones D. Function and mechanism of organic anion exudation from plant roots. Annu. Rev. Plant Biol. 52, 527–560 (2001). 10.1146/annurev.arplant.52.1.527 11337408

[b54] Haichar F. el Z., Santaella C., Heulin T. & Achouak W. Root exudates mediated interactions belowground. Soil Biol. Biochem. 77, 69–80 (2014).

[b55] Mommer L., Wagemaker C. A. M., De Kroon H. & Ouborg N. J. Unravelling below-ground plant distributions: A real-time polymerase chain reaction method for quantifying species proportions in mixed root samples. Mol. Ecol. Resour. 8, 947–953 (2008). 21585938 10.1111/j.1755-0998.2008.02130.x

[b56] Frostegard A. . Shifts in the structure of soil microbial communities in limed forests as revealed by phospholipid fatty acid analysis. Soil Biol. Biochem. 25, 723–730 (1993).

[b57] Bligh E. G. & Dyer W. J. A rapid method of total lipid extraction and purification. Can. J. Biochem. Physiol. 37, 911–917 (1959). 13671378 10.1139/o59-099

[b58] Loreau M. & Hector a. Partitioning selection and complementarity in biodiversity experiments. Nature 412, 72–6 (2001). 11452308 10.1038/35083573

[b59] Eisenhauer N., Bowker M. A., Grace J. B. & Powell J. R. From patterns to causal understanding: Structural equation modeling (SEM) in soil ecology. Pedobiologia (Jena). 58, 65–72 (2015).

[b60] Lefcheck J. S. piecewiseSEM: Piecewise structural equation modeling in R for ecology, evolution, and systematics. Methods Ecol. Evol. 7, 573–579 (2015).

