# Supplementary material for: Root biomass and exudates link plant diversity with soil bacterial and fungal biomass
Source: Sci Rep. 2017 Apr 4;7:44641. doi: 10.1038/srep44641 (PMC5379681; doi:10.1038/srep44641)
Supplement: Supplementary Information [file srep44641-s1.pdf]

Supporting Information

Root biomass and exudates link plant diversity with soil microbial communities

Nico Eisenhauer[1,2], Arnaud Lanoue[3], Tanja Strecker[4], Stefan Scheu[4], Katja Steinauer[1,2], Madhav P Thakur[1,2] & Liesje Mommer[5]

DNA analysis of root material

For preparation of reference standardsfor quantitative analysis of species-specific root biomass with real-time PCR, hand-made mixed ratios of all 6 species (1:1:1:1:1:1) were prepared from roots originating from the respective monocultures. For each species, the selected samples were pooled in a mortar containing liquid nitrogen and ground to obtain a homogenous sample. Each ground species was put in a 50 ml tube, with a hole pierced in the side to allow the evaporation of the liquid nitrogen and stored at -20°C until further processing. For each of the ten reference standards, 16.67 mg of each species was weighed in a 1.5 mL Eppendorf tube, using a micro balance (BL 120S, Sartorius, Göttingen, Germany), readable to 0.1 mg. During weighing samples were kept on ice as much as possible. The standards were stored at -20°C.

For DNA extraction,samples were first slightly defrosted and transferred from 2 ml into 1.5 ml Eppendorf tubes. After transfer, the samples were snap frozen in liquid nitrogen and further ground using glass rod attached to a hand mixer.The samples were eluted in two steps of 100µl elution buffer per step. The DNA concentration was measured using two different

methods, the Qubitfluorometer using the Quant-iTdsDNA HS Assay (Invitrogen) and the Nanodrop 1000 (Invitrogen). DNA concentrations of all samples were measured using the Nanodrop (OD 230/260/280).For the Qubit fluorometer, the provided protocol Quant-iT Assays was followed. The concentration was measured using the Quant-iTdsDNA HS option. The samples were diluted to obtain a DNA concentration of 0.5 ng μl^{-1} by adding an appropriate amount of MilliQ to the 10 µl sample.

Twenty-eight test samples in triplicate could be analyzed (28 x 3 = 84) on one plate for one species together with 4 reference standards in duplicate (8), a positive control sample in duplicate (2), containing only DNA of the target species, to ensure the correct primer pair was used and a no template control in duplicate (2, MilliQ). All samples were analyzed in triplicate, as the deviation between these technical replicates should be relatively low (note that these are no biological replicates).

Supplementary Table 1. Information on primers used for quantitative PCR of root samples.

Primer pairs 5'-3'	Direction	Sequence	Primer concentration (µM)
Centaurea jacea	Forward	CTCGCACATCCACGCACAC	2
	Reverse	TGCAGTGGTTTTCGTAGGAAGG	2
Anthoxanthum odoratum	Forward	TCATGTACTGTTGTACTGCGAAG	2
	Reverse	GAATCAAGCTGGACAGTAAATGAC	2
Festuca rubra	Forward	ACCGGAGATCGACAGCAAAACAG	4
	Reverse	TGTCCCTTGGTGGCGTTTTGG	4
Plantago lanceolata	Forward	GAGAAAGCAGTAGGAAACCACAGTG	4
	Reverse	GATCGAGATCTCTCACTCAAAACCCC	4
Rumex acetosa	Forward	CCTCGCCGTCTTCAAGAACT	4
	Reverse	CTCAGTTGCCGTTTTGTCCA	4
Phleum pratense	Forward	GCCCTCTTGATTTTCGCATC	4
	Reverse	CTCAGTTGCCGTTTTGTCCA	4

HPLC analysis root exudates

All microcosms were watered with 150 ml of deionized water, and with another 50 ml after 1.5 h. One micro suction cup was installed 1.5 h after watering in the center of each microcosm and 10 cm deep. A sterile 10 ml syringe generated low-pressure and collected soil solution. Soil solution (10 ml) was collected, transferred into sterile 15 ml falcons, immediately cooled, and stored until analyses at -20°C.

Root exudate solutions (10 ml) were filtered through a 0.22 µm filter (Millipore, Bedford, MA, USA), acidified to pH 2 with 1 N HCl, and partitioned against 5 ml ethyl acetate on an orbital shaker at 290 rpm (Bühler, Hechingen, Germany) for 1 h at room temperature. The organic phase was reduced to dryness under nitrogen and the residue taken up in 100 µl of methanol for high pressure liquid chromatography (HPLC) analyses. The HPLC apparatus consisted of a Waters system equipped with a gradient pump (Waters 600 controller, Milford, MA, USA), a cooled auto sampler (Waters 717 plus), and a UV–visible photodiode-array detector (Waters 996) set to acquire data from 200 to 400 nm. Analyses were performed on 3 µm column (250 × 4 mm, Multospher 120 RP18HP; CS-Service, Langerwehe, Germany) at 24°C. The mobile phase consisted of aqueous phosphoric acid (0.1% w/v; eluent A) and acetonitrile (eluent B) pumped at 0.5 mL min-1 into the HPLC system. The gradient started at 5% B and increased linearly to 72.5% in 60 min, followed by washing and reconditioning the column. Chromatogram peaks were identified according to their UV spectra with max plot detection (200-400 nm) by automated comparison with a library containing 200 plant natural products using Empower 2 software (Waters Corporation, Milford, MA, USA). Peaks that remained unidentified were not integrated. HPLC chromatograms from samples containing the same plant species were compared to establish a list of plant-specific root exudates. Based

on these automatic identifications, pure commercial standard solutions purchased from Sigma-Aldrich (St Louis, MI, USA) were prepared and injected in the HPLC apparatus for authentication of compound identity. For the calculations of root exudate amounts, quantification was done using 6-points calibration curves (1-100 µM) of pure reference standard of fumaric, 4-hydroxybenzoic, vanillic, and *p*-coumaric acids and then the concentrations were summed up.

Design of the experiment

The initial design of the experiment included an orthogonal manipulation of plant diversity and decomposer diversity (see Supplementary Table 2). However, as the decomposer treatments could not be successfully implemented (probably due to a dry period during the experiment), we focus on plant species richness effects in the present study and regard the decomposer treatments as potential source for variability in the dataset, which can be due to decomposer activity and/or decomposers dying and increasing the availability of organic matter.

Supplementary Table 2. Design of the experiment. Given are numbers of the microcosms (pot) with the respective plant diversity and decomposer diversity treatments as well as information on the presence of the different plant and decomposer species. Plant species: Fes: *Festucarubra*, Phl: *Phleumpratense*, Ant: *Anthoxanthumodoratum*, Pla: *Plantagolanceolata*, Cen: *Centaureajacea*, Rum: *Rumexacetosa*. Decomposer species: Lep: *Lepidocyrtuscyaneus*, Het: *Heteromurusnitidus*, Pro: *Proisotomaminuta*, cal: *Aporrectodea caliginosa*, ter: *Lumbricus terrestris*, rub: *Lumbricus rubellus*.

Pot	Plant species richness	Decomposer species richness	Ant	Fes	Phl	Cen	Pla	Rum	Lep	Het	Pro	cal	ter	rub
12	0	0												
28	0	0												
44	0	0												

Pot	Plant species richness	Decomposer species richness	Ant	Fes	Phl	Cen	Pla	Rum	Lep	Het	Pro	cal	ter	rub
3	1	1	1						1					
24	1	1	1							1				
25	1	1	1											1
62	1	1	1										1	
80	1	1	1									1		
81	1	1	1								1			
9	1	1		1										1
30	1	1		1									1	
48	1	1		1								1		
49	1	1		1							1			
67	1	1		1					1					
88	1	1		1						1				
8	1	1			1					1				
41	1	1			1									1
46	1	1			1								1	
64	1	1			1							1		
65	1	1			1						1			
83	1	1			1				1					
16	1	1				1						1		
17	1	1				1					1			
35	1	1				1			1					
56	1	1				1				1				
73	1	1				1								1
94	1	1				1							1	
19	1	1					1		1					
33	1	1					1				1			
40	1	1					1			1				
57	1	1					1							1
78	1	1					1						1	
1	1	1						1			1			
14	1	1						1					1	
32	1	1						1				1		
51	1	1						1	1					
89	1	1						1						1

Pot	Plant species richness	Decomposer species richness	Ant	Fes	Phl	Cen	Pla	Rum	Lep	Het	Pro	cal	ter	rub
4	1	3	1						1		1	1		
68	1	3	1						1	1		1		
36	1	3		1						1	1		1	
54	1	3		1						1	1	1		
22	1	3			1				1	1				1
86	1	3			1						1	1		1
52	1	3				1			1	1	1			
70	1	3				1					1		1	1
20	1	3						1	1		1			1
38	1	3						1	1	1			1	

Pot	Plant species richness	Decomposer species richness	Ant	Fes	Phl	Cen	Pla	Rum	Lep	Het	Pro	cal	ter	rub
58	1	6		1					1	1	1	1	1	1
74	1	6			1				1	1	1	1	1	1
26	1	6				1			1	1	1	1	1	1
10	1	6					1		1	1	1	1	1	1
95	1	6					1		1	1	1	1	1	1
42	1	6						1	1	1	1	1	1	1

Pot	Plant species richness	Decomposer species richness	Ant	Fes	Phl	Cen	Pla	Rum	Lep	Het	Pro	cal	ter	rub
59	3	1	1	1	1						1			
91	3	1	1	1		1				1				
27	3	1	1		1			1					1	
13	3	1	1			1	1				1			
61	3	1	1				1	1						1
75	3	1		1	1		1		1					
29	3	1		1	1			1	1					
11	3	1		1		1	1							1
45	3	1		1		1		1		1				
96	3	1		1		1		1				1		
43	3	1			1	1		1				1		
53	3	1				1	1	1				1		
77	3	1				1	1	1					1	
93	3	1				1	1	1				1		

Pot	Plant species richness	Decomposer species richness	Ant	Fes	Phl	Cen	Pla	Rum	Lep	Het	Pro	cal	ter	rub
55	3	3	1	1		1				1		1	1	
66	3	3	1	1				1	1		1		1	
34	3	3	1			1		1		1		1		1
50	3	3		1	1	1				1	1			1
84	3	3		1	1		1		1				1	1
82	3	3		1			1	1	1			1		1
18	3	3			1	1	1			1			1	1
2	3	3			1		1	1	1				1	1

Pot	Plant species richness	Decomposer species richness	Ant	Fes	Phl	Cen	Pla	Rum	Lep	Het	Pro	cal	ter	rub
60	3	6	1	1		1			1	1	1	1	1	1
90	3	6	1	1		1			1	1	1	1	1	1
15	3	6	1		1	1			1	1	1	1	1	1
47	3	6	1			1	1		1	1	1	1	1	1
63	3	6		1		1		1	1	1	1	1	1	1
79	3	6		1			1	1	1	1	1	1	1	1
31	3	6			1		1	1	1	1	1	1	1	1

Pot	Plant species richness	Decomposer species richness	Ant	Fes	Phl	Cen	Pla	Rum	Lep	Het	Pro	cal	ter	rub
92	6	0	1	1	1	1	1	1						
5	6	1	1	1	1	1	1	1		1				
21	6	1	1	1	1	1	1	1	1					
37	6	1	1	1	1	1	1	1			1			
69	6	1	1	1	1	1	1	1					1	
85	6	1	1	1	1	1	1	1						1

Pot	Plant species richness	Decomposer species richness	Ant	Fes	Phl	Cen	Pla	Rum	Lep	Het	Pro	cal	ter	rub
6	6	3	1	1	1	1	1	1			1	1	1	
7	6	3	1	1	1	1	1	1		1			1	1
23	6	3	1	1	1	1	1	1	1		1	1		
39	6	3	1	1	1	1	1	1			1	1	1	
71	6	3	1	1	1	1	1	1				1	1	1
87	6	3	1	1	1	1	1	1	1	1				1

Pot	Plant species richness	Decomposer species richness	Ant	Fes	Phl	Cen	Pla	Rum	Lep	Het	Pro	cal	ter	rub
76	6	6	1	1	1	1	1	1	1	1	1	1	1	1
